# Miscellaneous

**Published:** 1871

**Authors:** 


Article 241.—*A Case of Retro-Uterine Hæmatocele.** By Robert Barnes, M.D., F.R.C.P.

[British Medical Journal, October 1.]

Dr. Barnes described a case of retro-uterine hæmatocele, and illustrated it by a diagram constructed from the parts obtained by post-mortem examination. The case was remarkable by the blood-effusion having arisen from the rupture probably of a diseased ovary, and by its becoming completely encysted. The mass was of considerable size; it descended low into the pelvis, carrying the uterus forwards and upwards, so that the fundus was left during life projectiug over the symphysis pubis. The mass had caused great local distress, and symptoms of blood-infection arose. This induced resort to puncture of the cyst by the vagina. No improvement followed, and the patient sank. In reply to Dr. Tilt, the President said that the circulation of the blood was

*Abstract of a Paper read at the 38th annual meeting of the British Medical Association at Newcastle-on-Tyne.

circumscribed as the consequence of pelvic inflammation. Dr. Tilt said that, with regard to the treatment, the history of hæmatocele might be divided into two parts. The first part was that in which it was thought necessary to puncture with a large trocar, or make an incision, and take away as much of the blood as possible. The first pathologist who recognized these cases was Recamier, who did not describe them as hæmatocele, but as sanguineous pelvic tumors. His plan of treatment was to open them by incision, and to extract as much as possible. As a result, pyæmia and inflammation set in. He then made an injection, and the results were very favorable. A little later, the history of these cases was better gone into by Nelaton and Burnutz. Although they thought it was wrong to make any incision, they generally punctured. The mortality remained so considerable that they resolved to leave the cases to themselves—not to puncture, and sometimes to apply leeches either to the womb or the vagina. Dr. Gibson asked could there be pyæmia if there were no exposure? The President said that the blood was retained, and would sometimes undergo a change; the watery part of the blood might be absorbed, and so produce signs of pyæmia.—*Half-Yearly Abstract.*

---

Iodoform as a Neurotic.—Dr. Ephraim Cutler, of Boston, in the *Journal of the Gynæcological Society*, reports several cases of uterine disease, in which all nervous pains, etc., were successfully allayed by iodoform. Thus: "A married lady of about thirty-five years, suffering from ante-version, enlargement and ulceration of cervix, outside and in, could not sleep from the severe pain in the back, and nervous irritability of the whole system. This condition was much relieved and sleep procured by the nocturnal use of iodoform vaginal suppository." "A young man had a bleeding, irritable ulcer of leg, about two inches in diameter, and situated just above the external malcolus of the left leg. The hæmorrhage was considerable, and the pain so severe as to deprive of sleep. The hæmorrhage was checked by the persulphate of iron. Sulphate of morphia, gr. i, in lard ℥i, was applied to the surface, and bandages put on. Opium was given at night, and comparative comfort obtained. On applying iodoform, grs. xv to lard ℥i., to the surface, night and morning, the pain entirely ceased, and in three days the healing process was so rapid that more than one-half of the sore was covered by healthy cicatrix. I was astonished—could hardly credit it—but this did not alter the fact."—*Detroit Review of Medicine.*

Microscopists, who are constantly telling us wonderful stories, say that a cubic inch of tartar, such as collects on people's teeth, contains two hundred and fifty millions of animalcules. Bring us a tooth-brush.—*Ibid.*

On the Influence of Re-vaccination.—Dr. Rusnell, in the *Glascow Medical Journal* for May 1871, states that though there are thousands of re-vaccinated people in Glascow, the majority of the inhabitants having been re-vaccinated on account of the prevalence of small-pox, yet at a recent meeting of the Glascow Medico-Chirurgical Society not one member was able to adduce one single case of a re-vaccinated person having taken small-pox, and this is a universal experience. In times when small-pox prevails, every one above five, in an infected locality, ought to be re-vaccinated, irrespective of the quality of the primary vaccine marks; below five, these may be taken into consideration. And, in general, re-vaccination ought to be practiced universally on all persons above ten years of age.—*Edinburgh Medical Journal.*

On the Growth of the Nail as a Prognostic in Cerebral Paralysis.—Dr. S. Weir Mitchell, of Philadelphia, related before the College of Physicians, as reported in the *Am. Jour. of Med. Sciences*, that he had observed in several cases of palsy that the nails of the limbs of the affected side cease, on the occurrence of the accident, to grow. This he assured himself of by staining the nails at the roots with nitric acid. He was able to predict, on seeing after a time a white line of nail making its appearance, and before there were any other signs of improvement, that power was about to return to the limb, and that voluntary motion could shortly be restored.—*Druggist's Circular.*

Hydrocele of Round Ligament Mistaken for Hernia.—A case is reported in the *American Journal of Obstetrics*, in which the surgeon cut down extensively upon a supposed femoral hernia, in a female, which proved to be a hydrocele of the round ligament. The operator failed to recognize the nature of the tumor until, having exposed it fully and made divers efforts to reduce it, an exploring needle occasioned a jet of dropsical fluid, and the swelling vanished.—*Pacific Medical Journal.*

